# Advances in Purification of SARS-CoV-2 Spike Ectodomain Protein Using High-Throughput Screening and Non-Affinity Methods

**DOI:** 10.21203/rs.3.rs-778537/v1

**Published:** 2021-08-20

**Authors:** Nicole L. Cibelli, Gabriel F. Arias, McKenzie L. Figur, Shireen S. Khayat, Kristin M. Leach, Ivan Loukinov, Krishana C. Gulla, Daniel B. Gowetski

**Affiliations:** 1 Vaccine Research Center, National Institute of Allergy and Infectious Diseases, National Institutes of Health, Bethesda, Maryland 20892, USA.

**Keywords:** Biopharmaceutical development, cGMP, COVID-19, Downstream Processing, High-Throughput Screening, Ion Exchange Chromatography, Non-Affinity Chromatography, Manufacturing, SARS-CoV-2 Vaccine, Scale-Up, Viral Clearance

## Abstract

The spike (S) glycoprotein of the pandemic virus, SARS-CoV-2, is a critically important target of vaccine design and therapeutic development. A high-yield, scalable, cGMP-compliant downstream process for the stabilized, soluble, native-like S protein ectodomain is necessary to meet the extensive material requirements for ongoing research and development. As of June 2021, S proteins have exclusively been purified using difficult-to-scale, low-yield methodologies such as affinity and size-exclusion chromatography. Herein we present the first known non-affinity purification method for two S constructs, S_dF_2P and HexaPro, expressed in the mammalian cell line, CHO-DG44. A high-throughput resin screen on the Tecan Freedom EVO200 automated bioprocess workstation led to identification of ion exchange resins as viable purification steps. The chromatographic unit operations along with industry-standard methodologies for viral clearances, low pH treatment and 20 nm filtration, were assessed for feasibility. The developed process was applied to purify HexaPro from a CHO-DG44 stable pool harvest and yielded the highest yet reported amount of pure S protein. Our results demonstrate that commercially available chromatography resins are suitable for cGMP manufacturing of SARS-CoV-2 Spike protein constructs. We anticipate our results will provide a blueprint for worldwide biopharmaceutical production laboratories, as well as a starting point for process intensification.

## 1. Introduction

Following the emergence of the SARS-CoV-2 virus in late 2019, a platform approach to betacoronavirus spike protein stabilization in the pre-fusion conformation, along with early solved atomic-level structures of the stabilized spike, allowed for rapid selection of the SARS-CoV-2 spike protein as an antigen for vaccine development [1, 2]. Recombinant spike protein constructs, both full length and soluble ectodomain, are the basis of candidates in late-stage clinical trials, including those sponsored by Novavax, Sanofi Pasteur, and GSK [3, 4], and have the benefit of robust commercial experience and previous licensure. Thus, recombinant proteins are a worthwhile complement to the novel technologies in parallel development [5].

In addition to vaccine development, numerous efforts to produce large quantities of spike protein are underway in order to supply the high demand for therapeutic, diagnostic, and serosurveillance methods. In therapeutic monoclonal antibody development, standardization of binding assays is important for comparative data analysis. Spike protein binding assays are one method in use by the Coronavirus Immunotherapy Consortium for assessing antibody treatments [6]. Similarly, population-wide serological detection of SARS-CoV-2-specific antibodies with a spike protein ELISA is a useful tool for surveillance and containment, with throughput and cost benefits over PCR-based virus assays [7]. To supply these significant endeavors, a scalable, economical, rapid spike protein production protocol is of critical importance.

Various SARS-CoV-2 spike protein production cell types are currently in use and development, including insect [8, 9, 10], bacterial [11], and, predominantly, mammalian cell lines [12, 13, 14, 15, 16, 17, 18, 19]. Mammalian cell lines provide human- or human-like post-translational modifications, including glycosylation, but require longer culture durations to express protein [20]. Glycosylation around the receptor binding domain (RBD) of the spike protein is of specific interest, as it may play an important role in antibody recognition [21]. In early mammalian-cell based production runs of stabilized, soluble spike protein constructs, expression levels of 1 – 5 mg of protein per liter of Expi293 cell culture harvest were reported [13]. Yield optimization experiments, focusing mainly on transfection and cell culture conditions, have increased reported upstream titers to between 100 and 150 mg/L in CHO cells [17].

Importantly, all currently reported purification processes employ affinity resins, predominantly featuring immobilized metal affinity chromatography (IMAC) [9, 12, 13, 14, 15, 16, 17] and sometimes StrepTactin [17, 19, 18], lentil lectin [8, 9], immunoaffinity [22], or Anti-FLAG M2 [17] affinity chromatography. Except for lentil lectin, these methods require the inclusion of a tag in the sequence of the molecule and, generally, a protease-mediated cleavage step following purification. While these affinity methods yield a highly pure product and require little optimization or development work, they are difficult to scale to large manufacturing campaigns. Recently, advances have been made in affinity methods for application in cGMP environment, specifically in single-use applications, but cost, ligand supply chain complexities, and productivity remain a challenge [23, 24]. Additionally, when size-exclusion chromatography (SEC) is applied as a polish step after affinity chromatography [9, 13, 18], facility fit challenges arise; the required large column volumes and small load volumes necessitate an extra concentration step prior to chromatography or many cycles when manufactured at large scale.

To address these challenges, we employed cutting edge process development methods to find the conditions that enable inexpensive, high-yield purification using non-affinity resins suitable for large-scale manufacturing. Initial studies were performed using CHO-DG44 stable pools expressing the first reported stabilized ectodomain protein, named S_dF_2P, designed from the WA-01 viral sequence [1]. This construct consists of residues 1 – 1208 of the spike ectodomain, stabilized by two proline mutations in the S2 fusion machinery region. Additionally, the furin recognition motif, RRAR, at residues 682–685 was mutated to GSAS. In a recently reported Phase 1 clinical trial interim analysis, this construct adjuvanted with CpG 1018 and aluminum hydroxide has been shown to be well tolerated and immunogenic in healthy adults [25].

High-throughput chromatography resin screens using Tecan robotic liquid handlers and Repligen Robocolumns containing 0.1 mL of each respective resin were performed as previously described [26, 27, 28] to select lead candidates for process development. Additionally, to ensure a safety profile meeting regulatory agency guidance, viral clearance methods including low pH treatment and nanofiltration were screened for compatibility with the molecule and purification process [29, 30, 31]. In sum, a novel process utilizing non-affinity methods was developed in less than four calendar months.

To facilitate rapid product development, analytical and purification methods were developed simultaneously. Initial screening experiments utilized raw binding data, reported in nanometer shift, from the Octet platform. Later, a reference standard became available, and the Octet binding data were fit to a standard curve to report a product-specific concentration. Due to low pH interference with both Octet methods, product quantity was then inferred from GXII purity and A280 results for cation exchange (CEX) step development.

**Figure 1.**
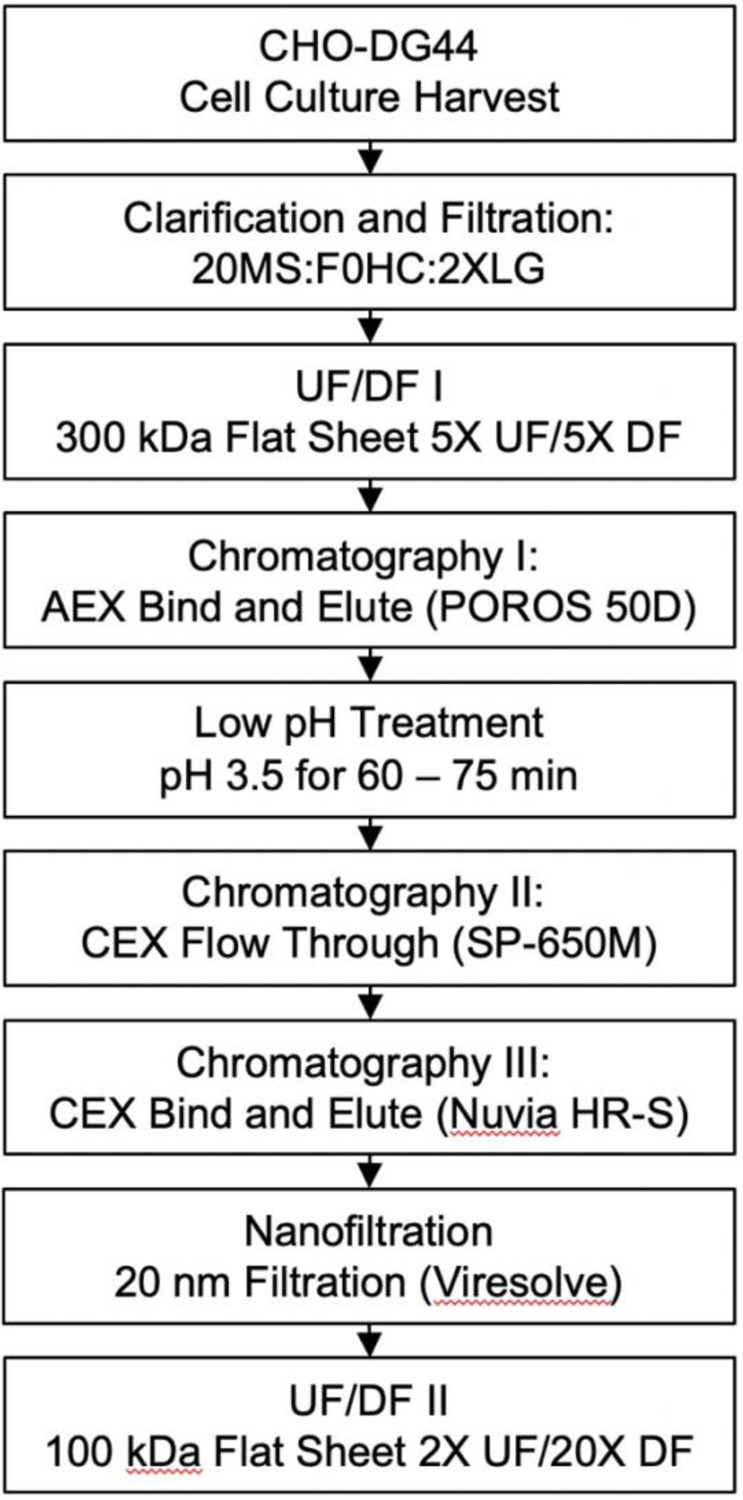
Process Flow Diagram for purification of stabilized S protein. 20MS: Clarisolve 20MS Depth Filter; F0HC: Millistak+ F0HC Depth Filter; 2XLG: Sartorius 2XLG 0.8/0.2 capsule filter; UF: ultrafiltration; DF: diafiltration; AEX: anion exchange; CEX: cation exchange.

The developed process, shown in Figure 1, consists of cell culture harvest clarification by depth filtration followed by ultrafiltration and diafiltration into a suitable buffer for anion exchange (AEX) capture chromatography. Following the AEX step, the material is titrated to pH 3.5 for low pH treatment and then subjected to a CEX step in flow through mode followed by another CEX step in bind-and-elute mode. The purified material is then subjected to nanofiltration and a final concentration/buffer exchange step.

Following process development, we applied the developed process with no further optimization to a CHO-DG44 stable pool expressing the recently reported stabilized construct, HexaPro, containing four additional proline mutations. The HexaPro construct was selected for the proof of concept run due to previous findings that “HexaPro expressed 9.8-fold higher than [S_dF_2P], had a ~5°C increase in Tm, and retained the trimeric prefusion conformation” [18]. The purification process and analytical methods were applied to the HexaPro stable pool, which yielded 163 mg of purified product per liter harvest.

The process described herein is scalable, cost-effective, and provides increased yields of highly pure, well-formed trimers. Moreover, these experiments provide a large dataset of commercially available chromatography resins for further exploration.

## 2. Results

### 2.1. Capture Resin Screen

The anion exchange resin screens yielded heat maps of S_dF_2P binding by mAb118 Octet, reported in raw nanometer shift, as well as total protein concentration by pathlength-corrected A280 (Figure S1). Importantly, the elution fractions between 100 mM NaCl and 500 mM NaCl showed variations in A280 signal. Generally, the Octet binding heat maps (Figure S1) show a significant portion of S_dF_2P in the flow through and chase fractions, potentially due to high loading density. When comparing S_dF_2P content to total A280, it is clear that successful separation is occurring as there are large A280 peaks but very low S_dF_2P content in fractions > 500 mM NaCl.

**Figure 2:**
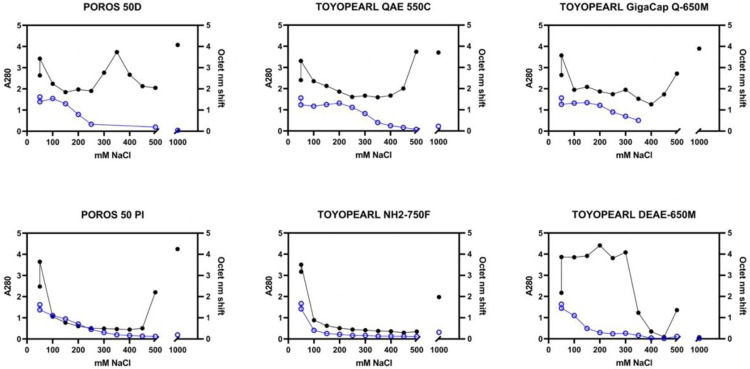
Example Resin Screen Data Analysis. Pathlength-corrected A280 (closed circles, black) and Octet binding data (open circles, blue) were plotted for increasing NaCl concentration elution fractions from 50 mM to 500 mM NaCl and, post-split, the 1000 mM NaCl strip for a selection of chromatography resins included in the pH 7 AEX resin screen. Top row: “hits” exhibited clear peaks in Octet binding < 500 mM NaCl and resolved peaks in A280 separately (either at varying NaCl concentration in the step elutions or in the 1000 mM NaCl strip), indicating successful purification. In contrast, resins not suited for capture, bottom row, showed various patterns, including gradient-like trailing with no clear peak (POROS 50 PI), overall lower Octet binding AUC (NH2–750F), or overlapping A280 peaks with no clear separation (DEAE-650M).

For a more detailed analysis, pseudo-chromatograms were created by plotting both A280 and Octet nm shift results from the pH 7.0 resin screen against NaCl concentration for each resin (Figure 2). Resins that had relatively narrow peaks with high AUC (Area Under Curve) in the Octet signal with good resolution from an A280 peak were considered lead candidates.

Pathlength-corrected A280 (closed circles, black) and Octet binding data (open circles, blue) were plotted for increasing NaCl concentration elution fractions from 50 mM to 500 mM NaCl and, post-split, the 1000 mM NaCl strip for a selection of chromatography resins included in the pH 7 AEX resin screen. Top row: “hits” exhibited clear peaks in Octet binding < 500 mM NaCl and resolved peaks in A280 separately (either at varying NaCl concentration in the step elutions or in the 1000 mM NaCl strip), indicating successful purification. In contrast, resins not suited for capture, bottom row, showed various patterns, including gradient-like trailing with no clear peak (POROS 50 PI), overall lower Octet binding AUC (NH2–750F), or overlapping A280 peaks with no clear separation (DEAE-650M).

Figure 2 also shows examples of candidates that were not selected, either for low Octet AUC, wide or trailing Octet curves, or overlap between the Octet and A280 peaks. Based on these analyses, in general, the pH 7.0 results indicated better separation than pH 8.0. POROS 50 D, QAE-550C, and GigacapQ 650M were selected for further optimization.

### 2.2. UF/DF I & Capture Resin Selection and Optimization

**Figure 3:**
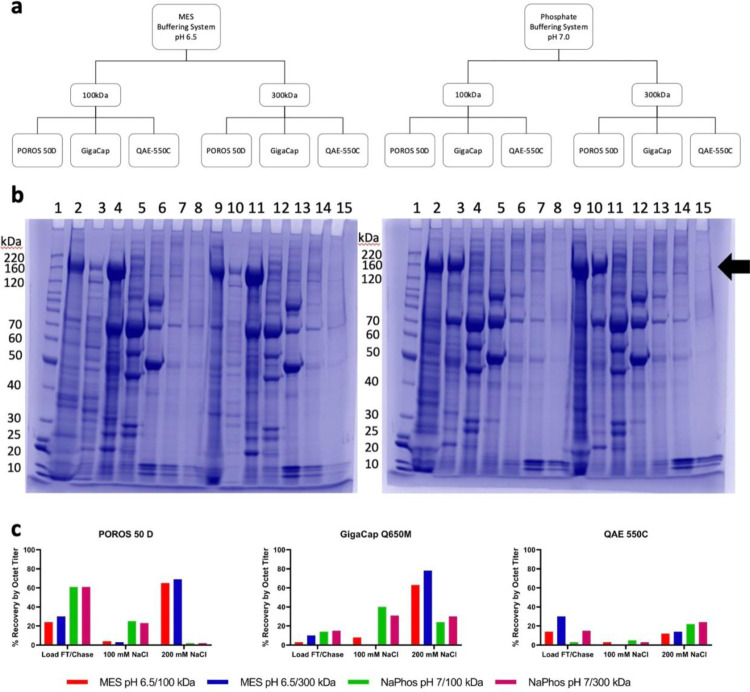
AEX Capture Step Resin Selection and Optimization. **(a)** Diagram depicting experimental design. **(b)** Representative SDS-PAGE from POROS 50 D. Left: MES pH 6.5. Right: Sodium Phosphate pH 7.0. In each gel 1: BenchMark Protein Ladder; 2 and 9: Load FT/Chase; 3 and 10: 100 mM NaCl; 4 and 11: 200 mM NaCl; 5 and 12: 300 mM NaCl; 6 and 13: 400 mM NaCl; 7 and 14: 500 mM NaCl; 8 and 15: 1000 mM NaCl. Lanes 2 – 8 in each gel: 100 kDa UF/DF I; Lanes 9 – 15: 300 kDa UF/DF I. **(c)** S_dF_2P recovery by product-specific octet titer in FT/Chase, 100 mM NaCl, and 200 mM NaCl fractions for each selected top resin.

Experimental factors such as buffer system, pH, and UF/DF I feed stream conditions were screened for impact on each candidate capture resin. First, the pH 7.0 buffer system used in the resin screen was compared to an MES pH 6.5 buffer system. The load material in each buffer system/pH combination was produced by both a 100 kDa UF/DF I membrane and a 300 kDa UF/DF I membrane to assess the impact of feed stream characteristics on capture step performance. Each chromatography run (Figure 3A) was subjected to an NaCl step gradient elution.

The elution fractions from the chromatography runs were assessed for recovery by octet titer and purity by HP-SEC and SDS-PAGE. Across all resins, the 300 kDa load material yielded an elution of higher purity than the 100 kDa load material (POROS 50D data shown in Figure 3). Furthermore, the pH 6.5 MES condition provided better resolution of the main S_dF_2P band from impurities in the flow through/chase than the pH 7.0 Sodium Phosphate condition, based on SDS-PAGE (Figure 3B). Thus, the 300 kDa-produced load material buffered in 25 mM MES, 25 mM NaCl pH 6.5 was selected.

All four runs on QAE-550C yielded overall low levels of the protein of interest compared to the other resins and was thus not considered for further optimization (Figure 3C). Both POROS 50 D and Gigacap Q650M had 200 mM NaCl elution fractions with about 50% purity by HP-SEC. By octet titer, these fractions yielded 69% and 78% recovery, respectively. The flow through/chase fraction for POROS 50 D contained 30% recovery, compared to 10% for Gigacap Q650M. Both resins had a negligible amount of S_dF_2P in the 100 mM NaCl fraction. Despite the higher recovery loss in the flow through/chase fraction, POROS 50 D was selected as the capture step because the total mass balance was closer to 100%, so modulation of residence time and loading density were paths forward to reduce loss in the flow through. Gigacap Q650M could also be chosen as a capture step to fit inventory or other laboratory-specific concerns.

Additionally, load material produced from a 300 kDa UF/DF I process, buffered in Sodium Phosphate or MES with 25 mM NaCl at pH 6.5 were assessed on POROS 50 D. MES was confirmed as the buffer system because the product band at ~200 kDa was more concentrated in fractions 7 through 10, compared to the Sodium Phosphate buffer system, where the band of interest was found in fractions 4 through 9 (Figure S3). The final process parameters can be found in Table S1.

### 2.3. Polish Step Resin Screen

**Figure 4:**
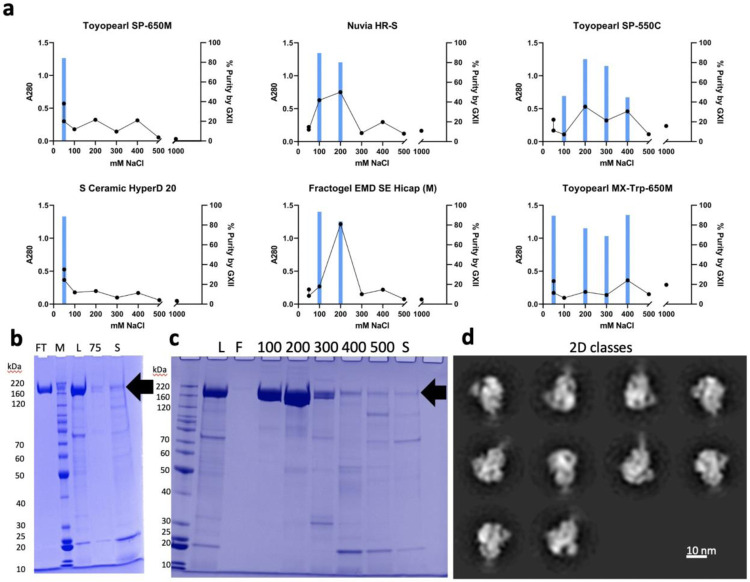
Cation Exchange Screen & Proof of Concept Results **(a)** Example subset of resin screen graphs with A280, black line/left axis, and percent purity by GXII, blue bar/right axis. First column: example candidate flow through chromatography resins. Second column: example bind and elute candidate resins. Third column: example low separation/broad product peak resins. **(b)** ToyoPearl SP-650M SDS-PAGE. FT: flow through; M: BenchMark Protein Ladder; L: Load; 75: 75 mM NaCl Wash; S: Strip. **(c)** Nuvia HR-S Bind and Elute SDS-PAGE. L: Load; FT: Flow through; 100 through 500: mM NaCl step gradient; S: strip. **(d)** TEM 2D Classes of Nuvia HR-S elution.

Due to interference with Octet titer at pH ≤ 5.0, the CEX polish resin screen samples were assessed for S_dF_2P content by using a concentration controlled GXII result. The purity of samples with an A280 greater than or equal to the median A280 value were reported to eliminate samples with high purity but unacceptably low yield. Additionally, total protein content as measured by corrected A280 were reported in the A280 heat map (Figure S2). The A280 and GXII results were plotted against NaCl concentration for each individual resin (examples shown in Figure 4A). The GXII method does not provide precise product-specific concentration values, but does provide a high-throughput purity measure compared to the time-intensive HP-SEC. For the fractions that show purity > 90%, yield is measured by A280.

The CEX screen results showed a few general patterns, shown in Figure 4A. First, numerous resins had high purity in the flow through and chase fractions (50 mM NaCl). These results indicate that a CEX step operated in flow through mode is viable for S_dF_2P polishing. Secondly, some resins showed high purity by GXII, high A280 fractions in NaCl fractions greater than 50 mM NaCl, indicating utility as a bind and elute polishing step. Lastly, some resins showed low overall A280 signal or a wide distribution of S_dF_2P fractions, which eliminated those resins from consideration for further development. Resins with concentrated S_dF_2P fractions and clear separation of other A280 signal were selected for further optimization: Tosoh Toyopearl SP-650M in flow through mode and BioRad Nuvia HR-S in bind and elute mode. The full resin screen data, shown in supplementary figure S2, provide ample data for further exploration as there were numerous fractions with purity by GXII greater than 80%.

### 2.4. Low pH treatment

Capture Step Eluate was titrated to pH 3.5 with 5N HCl and neutralized at incremental time points. Measuring mAb118 binding of each neutralized sample on the Octet platform relative to control, the relative binding was 96% after a 30-minute hold, 95% at 60 minutes, 89% at 90 minutes, and 99% at 120 minutes. These results indicate that low pH treatment for 60 minutes is a viable unit operation for implementation in a cGMP process when assessed by binding to mAb118.

### 2.5. Polish Step Selection and Optimization

POROS 50 D eluate was conditioned to 37.5 mM Sodium Citrate, 50 mM NaCl, pH 4.0 by dilution with 50 mM Sodium Citrate pH 4.0 and loaded onto the two selected cation exchange resins. Both SP-650M and Nuvia HR-S yielded a product pool that was about 85% pure based on HP-SEC and that were composed of well-formed trimers, as observed by NS-EM (example in figure 4D, full data in figure S4). SDS-PAGE of SP-650M shows a highly pure product in the flow through/chase at 50 mM NaCl, with no significant S_dF_2P population in the 75 mM NaCl fraction or strip (Figure 4B). The Nuvia HR-S step gradient elution SDS-PAGE indicates the fractions at 100 mM NaCl and 200 mM NaCl are enriched with the band of interest, with lower molecular weight species enriched in higher NaCl fractions (Figure 4C).

Experiments were performed to optimize run conditions such as pH and conductivity. For SP-650M, pH 4.0 provided a higher recovery by Octet Titer than pH 3.5 (82% vs. 51%, respectively, at a 22 mg/mL-r loading density) and was selected as the run condition. Nuvia HR-S elution buffer conductivity studies revealed a recovery > 80% across all elution conditions from 180 mM to 250 mM, with HCP levels increasing with NaCl concentration (figure S5). The Nuvia HR-S elution condition was set to 180 mM NaCl to minimize relative HCP while maintaining a high recovery.

Based on these experiments, Toyopearl SP-650M was chosen as a polish resin in flow through mode at 50 mM Sodium Citrate, 50 mM NaCl, pH 4.0. The flow through material from Toyopearl SP-650M was then loaded directly onto Nuvia HR-S and eluted at 50 mM Sodium Citrate, 180 mM NaCl pH 4.0. The two resins were selected to be operated in series to further reduce HCP levels.

### 2.6. 20 nm filtration

**Figure 5:**
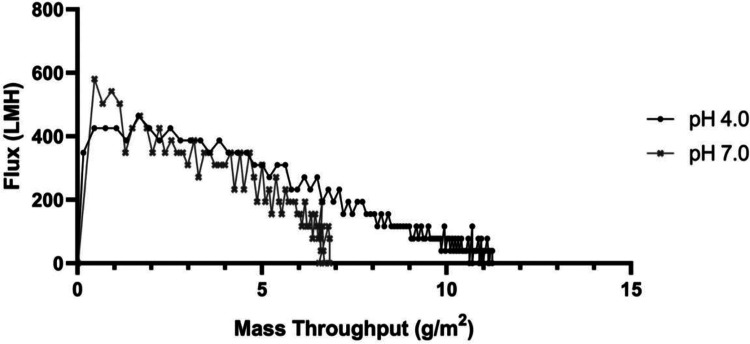
20 nm Filtration flux decay vs. mass throughput. Flux through the 20 nm filter is plotted against Mass throughput, measured by load A280 and volume.

Performance of the 20 nm filtration step, measured by flux decay, was assessed for both pH 4.0 and pH 7.0 operating conditions (Figure 5). This design space has been explored previously with regard to parvovirus clearance [32]. Both conditions showed adequate mass throughput, as measured by load A280, for selection and scale up in a cGMP process and either could be selected for fit into a process.

The Viresolve Shield Prefilter and Viresolve Pro Nanofilter at pH 4.0 were chosen as the pH condition for 20 nm filtration due to higher mass throughput. Although low pH treatment and 20 nm filtration are general industry practices, further experimentation such as live virus spike studies will be necessary to confirm viral inactivation and clearance for implementation into a cGMP process.

### 2.7. UF/DF II

Flat sheet membranes with 300 kDa and 100 kDa pore sizes were tested for UF/DF II. The 300 kDa membrane retentate contained no protein as measured by A280 and was therefore not analyzed further. The 100 kDa membrane was able to retain the protein, and the intermediate samples were assessed for HCP clearance. Peak HCP clearance, a 71-fold reduction, was identified to occur at the 20X DF sample point. The sample taken after the chase was pooled with the 20X DF material showed only a 17-fold reduction in HCP ppm from the load, so the chase was not pooled moving forward.

### 2.8. Proof of concept

**Figure 6:**
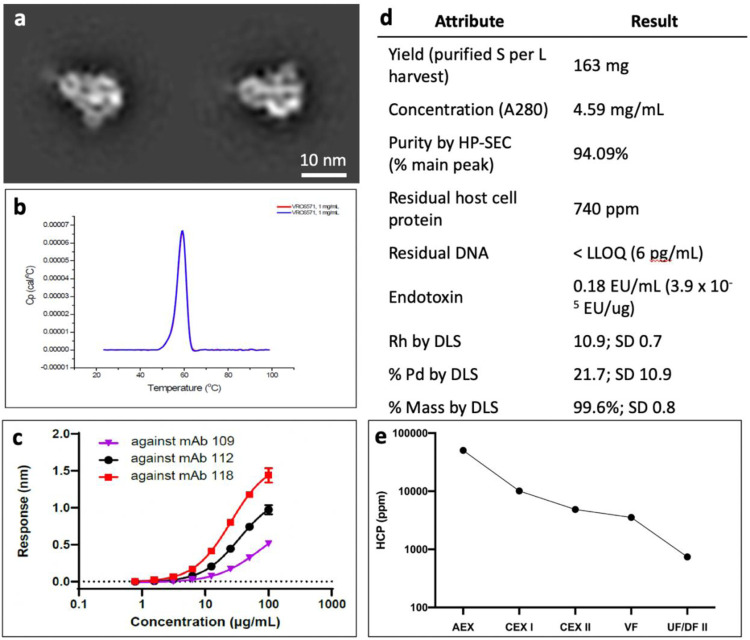
HexaPro characterization and process data. **(a)** NS-EM 2D Classes of purified HexaPro protein in 10 mM Histidine, 150 mM NaCl, 5% Sucrose (w/v) pH 6.5 **(b)** Differential Scanning Calorimetry in duplicate (overlapping curves) shows a Tm of 59.3°C (SD 0.1°C) **(c)** Octet binding to three SARS-CoV-2 spike-binding antibodies **(d)** Final material characterization data. Abbreviations include HCP: host cell protein; UF/DF: ultrafiltration/diafiltration; SEC: size exclusion chromatography; DLS: dynamic light scattering; Rh: hydrodynamic radius; % Pd: percent polydispersity. **(e)** Mean HCP (ppm) value (n = 2 except UF/DF II product n = 1) across purification unit operations.

The developed process, listed in Figure 1, was applied to the HexaPro construct. The upstream process in CHO-DG44 cells yielded 737.8 mg/L of HexaPro in day 14 cell culture as measured by Octet titer. The cell culture harvest was purified as described in the previous methods and yielded 163 mg of highly pure, well-formed trimer per liter of cell culture harvest for a 22% purification yield. The final product contained acceptable process-related impurity levels: 740 ppm HCP, and < 6 pg/mL (1.3 ppb) residual Host Cell DNA. Additional characterization data for the proof of concept run is displayed in Figure 6.

## 3. Discussion & Conclusion

To rapidly respond to the SARS-CoV-2 pandemic, large quantities of soluble, stabilized spike ectodomain protein are needed as a vaccine candidate and as a reagent for therapeutic and diagnostic development. To date, purification of such proteins has required costly and difficult-to-scale processes, including affinity and size-exclusion chromatography. This publication details the first known work to utilize high-throughput robotics to select commercially available, inexpensive chromatography media to purify coronavirus S proteins. We have demonstrated that the process presented herein is suitable for cGMP production of a next generation construct in addition to the construct for which it was developed. With this process serving as a backbone, SARS-CoV-2 S protein purification can be scaled up to serve the increasing demand to support ongoing clinical trials, therapeutic and diagnostic development, and, if necessary, future coronavirus vaccine development.

Previous reports have achieved a range of spike protein yields, usually less than 10 mg/L. Recent advances in transfection and cell culture conditions have increased upstream titers to 100–150 mg/L, but data is scarce on post-purification yields. Using the CHO-DG44 expression platform and the reported novel purification process for the HexaPro construct can yield as much as 737.8 mg/L in upstream expression and 163 mg/L of purified protein, an increase over all known reports.

The HexaPro product produced by the novel process was assessed by various analytical methods to be good quality with low levels of process- and product-related impurities. By DSC, the Tm of the HexaPro product was found to be 59.3°C, an increase over previously reported Tm for S_dF_2P [18]. Binding data, measured on the Octet platform, show differential binding curves to three SARS-CoV-2 specific antibodies (RBD-binding mAb109, S2-binding mAb112, and mAb118, which was utilized for all other Octet datasets herein and binds the NTD) [23]. Host cell protein was successfully cleared throughout each unit operation to a final level of 740 ppm.

The methods and datasets presented provide a strong basis for further optimization. The developed process should be assessed for purification of coronavirus spike proteins from divergent viral sequences, including the B.1.1.7 and B.1.351 variants [33, 34], spike proteins produced by different cell lines, and spike proteins with varying stabilizing and immune-targeting mutations, using the full resin screen results in the appendix as a starting point. Furthermore, there are clear areas for process intensification that will be of interest to the field. For instance, the ability to load the SP-650M flow through material directly onto Nuvia HR-S in bind and elute mode will enable implementation of continuous chromatography, providing additional efficiencies in scale up.

## 4. Materials and methods

### 4.1. Upstream

An expression vector encoding the gene for S_dF_2P or HexaPro along with a DHFR selection marker was transfected into CHO-DG44 cells by electroporation using the MaxCyte STX® scalable transfection system (MaxCyte, Gaithersburg, MD). Transfected cells were cultivated in an Multitron shaker (Infors HT, Switzerland) set to 37°C, 5% CO2, and 80% relative humidity with a shaking speed of 130 rpm (orbital throw of 1 inch) in CDM4CHO medium with 6 mM L-glutamine. Forty-eight hours after transfection, methotrexate (MTX) was added to the culture to a final concentration of 100 nM. Viable cell density and viability for the culture was assessed every three to four days using the Cedex HiRes (Roche CustomBiotech, Indianapolis, IN). Once a week, the cells were centrifuged at 100 × g for 10 minutes and resuspended in fresh CDM4CHO medium with 6 mM L-glutamine and 100 nM MTX. When the viability of the pools recovered to ≥ 80%, the medium was replaced with ActiCHO P medium containing 6 mM L-glutamine and 100 nM MTX.

### 4.2. Clarification and Concentration/Buffer Exchange

For harvest volumes less than 5 L, the harvest material was clarified of whole cells and cell debris by centrifugation at 3000 rpm for 30 minutes, followed by 0.8/0.2 μm sterile filtration (Sartorius Stedim, Germany). Alternatively, for larger volumes, the harvest was subjected to a depth filtration train consisting of Clarisolve 20MS followed by Millistak+ F0HC filters (MilliporeSigma, Burlington, MA) with a subsequent 0.8/0.2 μm sterile filter. The depth filters were arranged in series and equilibrated with 1X PBS. The cell culture harvest was pumped through the filters at a 60 LMH feed flux based on the F0HC filter area and chased with 1X PBS. Clarified harvest was stored at 2–8°C for further development activities.

### 4.3. UF/DF1

The clarified harvest was buffer exchanged using 100 kDa or 300 kDa Millipore Pellicon flat sheet membranes (MilliporeSigma, Burlington, MA) with a five-fold ultrafiltration and a five-fold diafiltration into various buffers as needed for capture chromatography. The feed flux was set to 330 LMH with a trans-membrane pressure of 10 psi. The 300 kDa flat sheet method was scaled up to a 1 m^2^ filter, with loading densities constant at around 10 L/m^2^.

### 4.4. Capture Resin Screen

Thirty-two anion exchange resins (Figure S1) were screened in duplicate at two pH conditions (pH 7.0 and pH 8.0) with a step gradient of NaCl elution conditions, in 50 mM NaCl increments ranging from 100 mM to 500 mM NaCl, followed by a 1 M NaCl strip. The resin screen was performed using the TECAN Evo system (TECAN group, Männedorf, Switzerland) in conjunction with robocolumns containing 0.1 mL of each resin (Repligen, Waltham, MA). Each column was loaded with concentrated, buffer exchanged harvest in 25 mM phosphate, 25 mM HEPES, 50 mM NaCl at either pH 7.0 or pH 8.0 to 222 mg/mL-r as measured by OD280 at a 2-minute residence time. The loading density was set to 222 mg/mL-r to ensure enough product would be loaded for analysis, based on an expected product titer ~20 mg/L. The elution fractions were collected in UV-transparent 96-well microplates (Corning, NY) and transferred to the in-line plate reader. The total protein content of each fraction was measured by pathlength-corrected A280 and the S_dF_2P content was measured by binding to a monoclonal antibody targeting the N-terminal domain on the Octet binding platform.

### 4.5. Capture Resin Selection and Optimization

Based on promising S_dF_2P binding and elution pattern data from the resin screen, resins were selected for further screening and development. Each resin was tested at pH 7.0 and pH 8.0 on an AKTA Avant (Cytiva, Picastaway, NJ), mimicking the process parameters from the resin screen (i.e., 2-minute residence time) with the loading density decreased to 50 g/L-r. All elution fractions were analyzed by SDS-PAGE. Following the initial screen, ToyoPearl QAE-550C (Tosoh Biosciences, King of Prussia, PA), POROS 50 D (ThermoFisher, Waltham, MA), and GigacapQ 650M (Tosoh Biosciences, King of Prussia, PA) were selected and further tested at pH 6.5 in an MES buffer system and pH 7.0 in a Sodium Phosphate buffer system to assess the impact of lower pH and buffer system on recovery (as measured by Octet titer) and purity (measured by HP-SEC) while including a head-to-head comparison to previous experiments performed in Sodium Phosphate pH 7.0. Subsequently, POROS 50 D was tested at pH 6.5 in both buffer systems listed above to investigate the impact each factor individually (i.e., buffer system and pH) (Figure S3).

### 4.6. Polish Resin Screen: CEX, HIC, and MM

Aliquots of POROS 50 D eluate were dialyzed using dialysis cassettes (ThermoFisher, Waltham, MA) into 50 mM Sodium Citrate, 50 mM NaCl pH 4.0 and pH 5.0. Thirty-one cation exchange resins (Figure S2), in duplicate, were loaded to 10 mg/mL-r by A280 measurement (1 OD = 1 mg/mL) for each pH condition with the same elution schema as the capture step resin screen. Due to low pH interference with the Octet titer assay, GXII was used to determine the purity of each fraction in addition to measuring total protein by pathlength-corrected A280. Fourteen hydrophobic interaction (HIC) and two mixed mode (MM) chromatography resins were evaluated at the robocolumn scale but did not yield promising separation based on SDS-PAGE (data not shown).

### 4.7. Polish Step Selection and Optimization: CEX

Two CEX resins were selected for AKTA-scale confirmation runs: Toyopearl SP-650M (Tosoh Biosciences, King of Prussia, PA) in flow through mode, and Nuvia HR-S (BioRad, Hercules, CA) in bind and elute mode based on high purity by GXII. AKTA-scale confirmation runs were analyzed via SDS-PAGE, purity by HP-SEC, and NS-EM. Toyopearl SP-650M optimization experiments included analyzing recovery by Octet titer at pH 3.5 vs. 4.0. Nuvia HR-S elution optimization experiments from 180–250 mM NaCl pH 4.0 were conducted to maximize recovery and HCP clearance (Figure S5). Elution fractions were analyzed by SDS-PAGE for purity, Octet titer for recovery, and HCP ELISA for HCP clearance.

### 4.8. 20 nm Filtration/Low pH treatment

Low pH treatment was evaluated for feasibility by holding process intermediate material at pH 3.5 for 30, 60, 90, and 120 minutes, followed by neutralization with 1 M Tris Base. The neutralized products were measured for binding on the Octet platform to assess any potential changes in antigenicity.

Nanofiltration performance was assessed by measuring flux and mass throughput on small scale, decoupled trains consisting of a Viresolve Shield or Shield H Prefilter and a 20-nm Viresolve Pro Filter (MilliporeSigma, Burlington, MA), run in constant pressure mode at 30 psi. A developmental lot of cation exchange-polished material was selected as the feed stream for 20 nm filtration, and either loaded directly at pH 4.0 or conditioned to pH 7.0 using 1 M Tris HCl, pH 8.0 prior to loading.

### 4.9. UF/DF II

Flat sheet membranes with 300 kDa and 100 kDa pore sizes (MilliporeSigma, Burlington, MA) were screened for final concentration, buffer exchange, and host cell protein (HCP) removal. Cation exchange chromatography elutions with high HCP (~250,000 ppm) were pooled from selection and optimization experiments and loaded onto 50 cm^2^ membranes. At a flux of 300 LMH and TMP of 7.3 psi, the material was concentrated two-fold and then diafiltered against 20 diavolumes of 10 mM Histidine, 150 mM NaCl, 5% Sucrose, pH 6.5. Samples of the retentate and permeate were taken at the end of ultrafiltration and at every five diavolumes. The filter was chased with one system volume of diafiltration buffer and the chase was pooled with the retentate for an additional sample point. Each fraction was analyzed by Octet titer for S_dF_2P-specific recovery, purity by HP-SEC, and residual HCP.

### 4.10. Proof of Concept

The developed process described in Figure 1 was applied to a CHO-DG44 stable pool harvest expressing the HexaPro stabilized spike construct. Cell culture harvest (6.5 L) was flowed through a depth filtration train consisting of one 0.11 m^2^ Clarisolve 20MS and one 0.11 m^2^ Millistak+ F0HC filter (MilliporeSigma, Burlington, MA) at 60 LMH, followed by 0.8/0.2 um sterile filtration (Sartorius Stedim, Germany). The clarified harvest was concentrated five-fold and then buffer exchanged against five diavolumes of 20 mM MES, 25 mM NaCl pH 6.5 using a 0.5 m^2^ 300 kDa flat sheet filter (MilliporeSigma, Burlington, MA). The buffer exchanged material was loaded onto POROS 50 D (ThermoFisher, Waltham, MA) at 20 – 25 mg/mL-r, and the elution, collected from 50 mAU – 80 mAU, was subjected to a 60-minute hold at pH 3.5. After low pH treatment, the material was diluted with 50 mM Sodium Citrate pH 4.0 to condition to the approximate equilibration conditions of the polish steps. The conditioned material was loaded onto Toyopearl SP-650M (Tosoh Bioscience, King of Prussia, PA) at < 15 mg/mL-r and chased with 5 CV of equilibration buffer. The flow-through and chase were pooled and loaded onto Nuvia HR-S (BioRad, Hercules, CA) at ~30 mg/mL-r, then eluted at 50 mM Sodium Citrate, 180 mM NaCl pH 4.0. Fractions of the Nuvia HR-S product were used for viral filtration studies, then the Nuvia HR-S product was pooled with the small scale aliquots of the 20 nm filtrate to forward process. The product pool was concentrated two-fold and buffer exchanged against 20 diavolumes of 10 mM Histidine, 150 mM NaCl, 5% Sucrose pH 6.5 on a 100 kDa flat sheet filter (MilliporeSigma, Burlington, MA).

### 4.11. Analytical Methods

#### 4.11.1. Octet

The binding assay was performed by biolayer interferometry (BLI) using Octet Red384 Instrument (FortéBio, Menlo Park, CA). For quantitative binding analysis of S_dF_2P (referred to as Octet titer), all reagents, calibrator, and samples are prepared by dilution in 1X kinetics buffer (KB) (FortéBio, Menlo Park, CA). The monoclonal antibody S652–118 (referred to as mAb118) (Vaccine Production Program, VRC, NIAID, NIH, Gaithersburg, MD) was immobilized onto a protein G biosensor (FortéBio, Menlo Park, CA), and followed by binding of S_dF_2P sample in a range of dilutions. The binding response is compared to a calibration curve of S_dF_2P of known concentrations. Serial dilutions of calibrator were performed at top of curve of 100 μg/mL scheme down to 0.78 μg/mL. Positive controls were in the form of a spike sample prepared in 1X KB at 40 μg/mL and diluted to 2X, 4X, and 8X, also in 1X KB. Each sample was diluted into the linearity range of the assay. The mAb118 stock was diluted to a concentration of 10 μg/mL. Four steps of assay include: (1) regeneration: 5 sec × 3 cycles with 500 mM phosphoric acid and 1X KB; (2) loading: 120 s with mAb118; (3) baseline: 30 s with 1X KB; (4) associate: 120 s with sample. The %CV for the calibration standard curve replicates was ≤ 20% for all points above 3.1 μg/mL. 4PL curve fit R2 was > 0.98. The recovery of spike was in a range of 80–120%.

For full curve binding analysis of S_dF_2P with mAb118, all reagents, calibrator, and samples are prepared by dilution in 1X PBS (Lonza). Serial dilutions of S_dF_2P sample and calibrator were performed at top of curve of 100 μg/mL scheme down to 0.78 μg/mL and a zero. The assay consisted of five steps: (1) regeneration: 5 sec × 3 cycles with 500 mM phosphoric acid and 1X KB; (2) baseline: 60 s with 1X PBS; (3) loading 180 s with mAb 118; (4) baseline: 60 s with 1X PBS; (5) association: 180 s with sample diluted serially in 1X PBS. The resulting data were fit to a 1:1 binding model. The %CV of response values for all sample and calibrator replicates was ≤ 20% for all points above 0.78 μg/mL.

#### 4.11.2. GXII

Four microliters of sample were mixed with 16 μL of reducing buffer (a mixture of SDS, LDS, and DTT) and denatured at 90°C for 5 minutes. Samples were allowed to cool to room temperature prior to the addition of 4 μL of dye. The samples were covered in foil, vortexed, and left to incubate in the dark for 1 hour. The dye reaction was quenched with 210 μL of stop solution and 105 μL of the labeled protein was loaded into a GXII plate. The plate was loaded into the instrument and run using the HT Pico Protein Express 200 Programming (PerkinElmer, Waltham, MA).

#### 4.11.3. High Performance Size-exclusion Chromatography (HP-SEC)

HP-SEC is a method where molecules are separated by size, specifically their hydrodynamic radius, and in this case detected through fluorescence (FLR). The S_dF_2P product purity is assessed using the SRT 500A SEC column (Sepax, Newark, DE) by FLR detection at excitation wavelength 280 nm and emission wavelength at 348 nm. The S_dF_2P purity is determined by the percent area of the main peak, while the S_dF_2P aggregation is determined by the percent area of the high molecular weight species and smaller proteins are eluted as the lower molecular weight species. The approximate molecular weight can also be determined with HP-SEC by comparing it with the gel filtration standard (GFS). The retention times of each peak that correspond to various molecular weights of the GFS can then be compared with the S_dF_2P main peak with an overlay of the chromatograms, which determined that the S_dF_2P main peak (S_dF_2P glycoprotein) is greater than 670 kDa.

#### 4.11.4. Host Cell Protein (HCP)

The CHO HCP assay is a two-site immunoenzymetric assay (Cygnus Technologies, Oakton, VA). Samples containing CHO HCPs are reacted simultaneously with a horseradish peroxidase (HRP) enzyme labeled anti-CHO antibody (goat polyclonal) in microtiter strips coated with an affinity purified capture anti-CHO antibody. The immunological reactions result in the formation of a sandwich complex of solid phase antibody-HCP-enzyme labeled antibody. The microtiter strips are washed to remove any unbound reactants. The substrate, tetramethylbenzidine (TMB) is then reacted. The amount of hydrolyzed substrate is read on a microtiter plate reader and is directly proportional to the concentration of CHO HCPs present.

#### 4.11.5. Host Cell DNA (HCD)

The residual CHO HCD assay kit (ThermoFisher, Waltham, MA) employs both a DNA extraction procedure and a QPCR quantitation procedure. CHO DNA extraction is performed utilizing the semi-automated MagMAX extraction method with the PrepSEQ Residual DNA Sample Preparation system. QPCR quantitation of residual DNA is performed utilizing the resDNASEQ Human Residual DNA Quantitation System. The primers and Taqman probe of the assay are highly specific, detecting only a hamster-specific region of a multicopy genetic element, with no cross-reactivity with unrelated DNA. The broad linear range of the QPCR assay allows for the testing of samples with variable levels of Human DNA in the sample assay, with a lower limit of quantitation (LLOQ) of 6 pg/mL.

#### 4.11.6. A280

Unless otherwise stated, concentration was determined by measuring absorbance at 260 nm, 280 nm, 340 nm, 900 nm, and 975 nm and using the pathlength correction displayed in equation 1 for high-throughput experiments. For lab-scale optimization experiments, absorbance at 280 nm was coupled with the empirically determined extinction coefficient of 1.00 for concentration measurement.


Equation 1. A=0.173*(A280−A340)/(A975−A900)

#### 4.11.7. Negative-stain electron microscopy

For protein preparations at neutral pH, the sample was diluted to 0.02 mg/ml with 10 mM HEPES, pH 7.4, supplemented with 150 mM NaCl. For protein preparations at acidic pH, 10 mM sodium-acetate supplemented with 150 mM NaCl was used instead, with the pH of the dilution buffer matching that of the sample. A 4.7-μL drop of the diluted sample was placed on a glow-discharged carbon-coated copper grid (CF200-Cu, Electron Microscopy Sciences, Hatfield, PA) for 15 s. The drop was then removed with filter paper, and the grid was washed by applying consecutively three 4.7-μL drops of the buffer used for dilution in the same manner. Negative staining of protein molecules adsorbed to the carbon layer was performed by applying consecutively three 4.7-μL drops of 0.75% uranyl formate in the same manner, and the grid was air-dried. Datasets were collected using an FEI T20 transmission electron microscope (Chalmers, Gothenburg, Sweden) operated at 200 kV and equipped with an Eagle CCD camera. The nominal magnification was 100,000x, corresponding to a pixel size of 2.2 Å, and the defocus was set at −1.0 μm. Data was collected automatically using SerialEM [35]. Particles were picked from the micrographs automatically using in-house written software (YT, unpublished). 2D classification was performed using Relion 1.4 [36].

#### 4.11.8. SDS-PAGE

SDS-PAGE were performed using ThermoFisher Scientific (Waltham, MA) materials, including Bolt^™^ 4–12% Bis-Tris Plus gels and a running buffer of 1X MOPS. All samples were subjected to NuPage reducing agent and diluted in Bolt 4X LDS sample buffer prior to loading. BenchMark Protein ladder was used as a molecular weight reference for each gel. Each gel was subjected to 150 V for 55 minutes, rinsed with DI water, and then stained with GelCode Blue Safe protein stain.

#### 4.11.9. DLS and DSC

DLS and DSC methods were performed as previously reported [37].

## Supplementary Material

Supplement 1

## Abbreviations:

AEX: Anion exchange
CEX: cation exchange
VF: viral filtration
VI: viral inactivation
TFF: tangential flow filtration
UF: ultrafiltration
DF: diafiltration
