# Supplementary material for: Advances in Purification of SARS-CoV-2 Spike Ectodomain Protein Using High-Throughput Screening and Non-Affinity Methods
Source: Res Sq. 2021 Aug 20:rs.3.rs-778537. Preprint. [Version 1] doi: 10.21203/rs.3.rs-778537/v1 (PMC8382130; doi:10.21203/rs.3.rs-778537/v1)
Supplement: Supplement 1 [file 8b43c3f9b95cf2ad930c9bcd.pdf]

Resin	Flowthrough	Chase	100 mM NaCl	150 mM NaCl	200 mM NaCl	250 mM NaCl	300 mM NaCl	350 mM NaCl	400 mM NaCl	450 mM NaCl	500 mM NaCl	Strip
Q Ceramic HyperD 20	2.753	3.662	1.171	1.258	1.733	1.511	1.013	1.114	0.767	1.541	3.125	4.496
Q Ceramic HyperD F	2.436	3.710	0.658	0.789	1.192	0.997	0.817	0.788	0.836	2.417	3.085	4.052
Q Hypercel	2.175	3.656	0.658	0.924	1.069	2.783	4.533	2.367	0.983	0.664	0.378	0.787
MacroPrep DEAE	2.251	3.512	1.979	1.632	1.776	2.159	3.396	3.008	2.222	1.557	2.168	3.667
MacroPrep High Q	2.308	3.384	2.335	1.935	1.774	1.498	1.696	1.414	2.694	3.795	2.750	3.949
Nuvia Q	2.502	3.546	1.609	2.123	2.251	1.776	2.041	1.801	1.473	2.036	2.888	4.427
UnoSphere Q	1.855	3.437	1.189	1.552	1.498	1.243	1.370	2.245	2.629	1.659	0.738	1.356
ANX Sepharose 4FF High Sub	2.065	3.811	1.992	1.735	1.569	1.457	1.747	2.262	3.434	3.369	2.288	4.019
Capto DEAE	2.078	3.543	0.956	1.110	1.106	1.041	1.035	1.442	2.684	2.672	1.803	2.358
Capto Q	2.155	3.552	1.030	1.224	1.208	1.132	1.160	1.141	1.790	2.557	2.213	3.697
DEAE Sepharose FF	2.241	3.533	2.147	1.928	2.011	2.328	3.756	3.482	1.652	0.988	0.756	0.475
Q Sepharose XL	1.954	3.530	0.846	0.951	0.979	0.776	0.830	0.820	0.859	1.523	2.276	4.140
Source 30Q	2.475	3.786	2.259	1.873	1.928	2.367	3.425	3.265	2.098	1.027	1.893	2.914
Eshmuno Q	2.246	3.461	1.191	1.441	1.238	1.020	1.186	1.376	2.548	2.487	1.356	2.557
Fractogel EMD DEAE (M)	2.349	3.563	1.984	1.918	1.965	1.950	2.255	2.529	2.394	1.874	1.938	3.829
Fractogel EMD TMAE (M)	2.280	3.720	3.456	2.388	2.195	2.293	3.108	3.246	2.240	1.349	1.154	3.824
Fractogel EMD TMAE Hicap (M)	2.671	3.453	2.329	2.687	2.219	2.177	2.144	1.570	1.777	2.893	1.268	3.982
Hypercel Star AX	2.302	3.198	0.465	0.396	0.439	0.438	0.427	0.372	0.378	0.383	1.544	4.390
POROS 50D	2.637	3.429	2.241	1.843	1.973	1.901	2.764	3.736	2.668	2.126	2.047	4.071
POROS 50 HQ	2.796	3.445	2.704	2.103	1.835	1.565	1.496	1.499	1.867	2.458	1.536	3.284
POROS XQ	2.406	3.200	1.015	1.520	1.662	1.453	1.502	1.219	1.666	2.608	1.485	4.115
POROS 50 PI	2.479	3.646	1.057	0.773	0.607	0.512	0.495	0.470	0.445	0.508	2.206	4.246
TOYOPEARL NH2-750F	3.174	3.506	0.887	0.621	0.512	0.443	0.413	0.375	0.360	0.295	0.347	1.977
TOYOPEARL DEAE-650M	2.174	3.864	3.853	3.916	4.409	3.814	4.081	1.230	0.347	0.086	1.355	0.073
TOYOPEARL DEAE-650C	2.117	3.835	3.736	4.290	4.139	3.457	1.873	0.442	0.107	0.058	0.046	0.064
TOYOPEARL GigaCap Q-650M	2.648	3.578	1.950	2.089	1.874	1.745	1.952	1.526	1.265	1.736	2.720	3.896
TOYOPEARL GigaCap Q-650S	3.037	3.493	2.456	2.380	2.112	2.117	2.450	1.921	1.609	1.464	2.532	3.786
TOYOPEARL SuperQ-650C	2.186	3.418	1.599	1.688	1.759	1.614	2.884	4.105	2.391	0.958	0.506	1.300
TOYOPEARL SuperQ-650M	2.328	3.612	2.197	2.164	2.100	1.833	1.993	4.242	3.200	1.298	0.646	2.018
TOYOPEARL QAE 550C	2.400	3.310	2.352	2.129	1.857	1.606	1.665	1.591	1.668	2.006	3.743	3.706
TOYOPEARL Q-600C AR	2.105	3.535	0.884	0.821	0.712	0.541	0.482	0.418	0.327	0.266	0.391	4.412
TOYOPEARL GigaCap DEAE-650M	2.139	3.629	1.968	1.945	1.838	1.927	1.827	1.986	3.288	3.533	2.599	4.184

Resin	Flowthrough	Chase	100 mM NaCl	150 mM NaCl	200 mM NaCl	250 mM NaCl	300 mM NaCl	350 mM NaCl	400 mM NaCl	450 mM NaCl	500 mM NaCl	Strip
Q Ceramic HyperD 20	1.489	1.231	0.702	0.921	1.170	0.717	0.333	0.181	-0.042	0.140	NR	NR
Q Ceramic HyperD F	1.574	1.248	0.506	0.787	1.124	0.616	0.293	0.145	0.112	0.085	0.067	0.126
Q Hypercel	1.564	1.264	0.460	0.376	0.300	0.199	0.196	0.149	0.141	0.102	0.066	0.082
MacroPrep DEAE	1.632	1.508	1.333	0.888	0.505	0.273	0.237	0.191	0.201	0.186	0.156	0.157
MacroPrep High Q	1.652	1.411	1.493	1.254	0.883	0.423	0.284	0.193	0.162	0.108	0.073	0.217
Nuvia Q	1.607	1.267	1.223	1.409	1.433	1.101	0.797	0.471	0.291	0.155	0.120	0.159
UnoSphere Q	1.580	1.244	0.872	1.132	1.034	0.598	0.364	0.155	0.078	0.047	0.039	0.090
ANX Sepharose 4FF High Sub	1.604	1.368	1.150	1.198	1.278	1.037	0.575	0.235	0.178	0.123	0.090	0.172
Capto DEAE	1.450	1.142	0.481	0.547	0.489	0.401	0.257	0.153	0.114	0.065	NR	NR
Capto Q	1.552	1.238	0.646	0.662	0.524	0.317	0.219	0.131	0.105	0.061	0.047	0.056
DEAE Sepharose FF	1.516	1.297	1.061	1.116	0.906	0.389	0.174	0.093	0.061	0.061	0.029	0.021
Q Sepharose XL	1.570	1.261	0.504	0.622	0.719	0.536	0.332	0.189	0.100	0.063	0.056	0.036
Source 30Q	1.584	1.449	1.369	1.119	0.568	0.218	0.141	0.086	0.075	0.084	0.041	0.049
Eshmuno Q	1.566	1.246	0.804	1.034	1.081	0.726	0.395	0.165	0.084	0.053	0.032	0.037
Fractogel EMD DEAE (M)	1.553	1.367	1.148	0.941	0.737	0.464	0.263	0.179	0.149	0.128	0.091	0.053
Fractogel EMD TMAE (M)	1.592	1.470	1.251	1.228	0.761	0.404	0.196	0.115	0.094	0.088	0.070	0.128
Fractogel EMD TMAE Hicap (M)	1.604	1.271	1.310	1.452	1.279	0.814	0.429	0.222	0.166	0.090	0.339	0.201
Hypercel Star AX	1.596	1.207	0.373	0.381	0.396	0.342	0.330	0.276	NR	NR	NR	NR
POROS 50D	1.620	1.395	1.542	1.298	0.795	0.331	NR	NR	NR	NR	0.196	0.043
POROS 50 HQ	1.613	1.446	1.570	1.441	1.258	0.873	NR	NR	NR	NR	NR	NR
POROS XQ	1.572	1.186	0.963	1.241	1.121	0.922	0.846	0.568	0.285	0.166	0.116	0.236
POROS 50 PI	1.619	1.378	1.108	0.947	0.706	0.451	0.307	0.198	0.164	0.137	0.123	0.197
TOYOPEARL NH2-750F	1.667	1.417	0.405	0.255	0.215	0.166	0.164	0.130	0.130	0.123	0.114	0.313
TOYOPEARL DEAE-650M	1.634	1.445	1.096	0.491	0.295	0.240	0.263	0.164	0.041	0.013	0.113	0.019
TOYOPEARL DEAE-650C	1.590	1.421	0.961	0.439	0.252	0.231	0.178	0.048	0.020	0.011	0.435	0.009
TOYOPEARL GigaCap Q-650M	1.563	1.265	1.321	1.344	1.219	0.904	0.703	0.505	NR	NR	NR	NR
TOYOPEARL GigaCap Q-650S	1.613	1.323	1.425	1.357	1.159	0.916	NR	NR	NR	NR	0.376	0.000
TOYOPEARL SuperQ-650C	1.586	1.255	1.208	1.162	0.798	0.437	NR	NR	NR	NR	NR	NR
TOYOPEARL SuperQ-650M	1.559	1.269	1.423	1.210	0.617	0.271	0.177	0.111	0.102	0.110	0.067	0.068
TOYOPEARL QAE 550C	1.557	1.237	1.167	1.247	1.316	1.113	0.821	0.396	0.249	0.166	0.073	0.221
TOYOPEARL Q-600C AR	1.539	1.247	0.506	0.642	0.815	0.545	0.295	0.156	0.107	0.071	0.053	0.080
TOYOPEARL GigaCap DEAE-650M	1.575	1.247	1.298	1.281	1.077	0.836	0.603	0.503	0.429	0.264	0.158	0.264

LEGEND: NR | LOW | HIGH

Resin	Flowthrough	Chase	100 mM NaCl	150 mM NaCl	200 mM NaCl	250 mM NaCl	300 mM NaCl	350 mM NaCl	400 mM NaCl	450 mM NaCl	500 mM NaCl	Strip
Q Ceramic HyperD 20	5.035	2.970	0.828	1.162	1.466	1.395	1.003	0.966	0.647	1.433	2.669	4.080
Q Ceramic HyperD F	4.766	3.212	0.646	0.829	1.096	1.061	0.906	0.709	0.847	1.886	2.310	3.631
Q Hypercel	4.641	2.832	0.801	1.239	1.499	2.712	3.773	1.631	0.805	0.596	0.392	1.081
MacroPrep DEAE	4.666	3.139	1.501	1.335	1.763	2.205	2.503	1.824	1.509	1.404	2.403	3.455
MacroPrep High Q	4.858	2.906	1.606	1.587	1.547	1.420	1.639	1.436	2.490	3.026	1.833	3.440
Nuvia Q	5.109	2.649	1.054	1.700	2.003	1.663	1.910	1.649	1.473	1.785	2.372	4.247
UnoSphere Q	4.790	2.959	0.857	1.334	1.430	1.167	1.351	2.049	2.134	1.263	0.558	1.087
ANX Sepharose 4FF High Sub	4.663	3.150	1.432	1.483	1.531	1.603	1.791	2.295	3.077	2.514	1.798	4.179
Capto DEAE	4.886	2.922	0.725	1.066	1.211	1.060	1.362	2.057	2.626	1.765	0.969	1.769
Capto Q	4.706	2.890	0.780	1.056	1.182	1.218	1.168	1.108	1.551	2.105	1.878	3.421
DEAE Sepharose FF	4.729	3.171	2.068	2.346	3.107	3.572	3.086	1.556	0.615	0.292	0.139	0.149
Q Sepharose XL	4.635	2.843	0.521	0.663	0.768	0.750	0.810	0.681	0.756	1.200	1.819	3.693
Source 30Q	4.935	3.325	1.811	1.610	1.815	2.120	2.770	2.597	1.714	0.837	1.417	2.792
Eshmuno Q	4.807	2.759	0.962	1.279	1.207	1.037	1.134	1.360	2.313	1.916	1.099	1.794
Fractogel EMD DEAE (M)	4.852	3.297	1.969	1.833	1.932	1.805	2.342	2.374	2.546	1.845	1.308	2.728
Fractogel EMD TMAE (M)	4.785	3.441	2.980	2.155	2.161	2.060	2.937	2.680	1.723	1.082	0.865	1.551
Fractogel EMD TMAE Hicap (M)	5.040	2.894	1.721	2.457	2.199	2.162	1.995	1.484	1.694	2.370	0.889	4.345
Hypercel Star AX	4.969	2.809	0.334	0.335	0.378	0.368	0.362	0.331	0.350	0.348	1.235	4.291
POROS 50D	4.793	3.018	1.667	1.581	1.854	1.998	2.999	2.663	2.071	1.370	1.662	3.403
POROS 50 HQ	4.996	3.076	2.241	1.922	1.819	1.562	1.554	1.578	2.153	2.170	1.100	1.492
POROS XQ	4.949	2.934	0.777	1.545	1.632	1.481	1.451	1.073	1.507	2.180	1.162	4.039
POROS 50 PI	4.963	1.087	1.071	0.938	0.957	0.761	0.788	0.729	0.732	0.862	0.992	4.481
TOYOPEARL NH2-750F	4.925	3.297	0.552	0.421	0.355	0.354	0.539	0.486	0.420	0.491	0.732	1.975
TOYOPEARL DEAE-650M	4.763	3.519	3.315	3.769	4.264	2.964	3.573	1.293	0.296	0.085	0.465	0.098
TOYOPEARL DEAE-650C	4.525	3.416	3.500	3.749	3.734	2.677	1.884	0.435	0.118	0.063	0.049	0.081
TOYOPEARL GigaCap Q-650M	4.960	2.642	1.136	1.650	1.663	1.570	1.780	1.320	1.135	1.480	2.149	1.978
TOYOPEARL GigaCap Q-650S	4.989	2.653	1.741	2.069	1.939	1.835	2.239	1.649	1.398	1.241	2.011	1.677
TOYOPEARL SuperQ-650C	4.622	2.938	1.089	1.382	1.589	1.366	2.345	3.112	1.626	0.640	0.358	1.073
TOYOPEARL SuperQ-650M	4.971	3.140	1.558	1.752	1.872	1.411	1.787	3.263	2.179	0.823	0.435	1.394
TOYOPEARL QAE 550C	4.288	1.450	0.775	0.947	0.879	0.721	0.804	0.799	0.777	0.882	1.567	2.088
TOYOPEARL Q-600C AR	4.837	2.828	0.764	0.793	0.770	0.613	0.511	0.468	0.377	0.329	0.571	4.134
TOYOPEARL GigaCap DEAE-650M	4.969	2.929	1.519	2.262	2.430	2.215	2.975	3.961	2.884	1.399	0.857	2.024

Resin	Flowthrough	Chase	100 mM NaCl	150 mM NaCl	200 mM NaCl	250 mM NaCl	300 mM NaCl	350 mM NaCl	400 mM NaCl	450 mM NaCl	500 mM NaCl	Strip
Q Ceramic HyperD 20	1.506	1.291	0.899	1.083	1.254	1.026	0.517	0.294	0.291	0.215	0.185	0.176
Q Ceramic HyperD F	1.521	1.342	0.813	0.926	1.182	0.959	0.609	0.325	0.234	0.155	0.150	0.166
Q Hypercel	1.534	1.258	0.823	0.396	0.315	0.213	0.205	0.173	0.174	0.148	0.127	0.126
MacroPrep DEAE	1.444	1.396	1.194	0.562	0.272	0.197	0.250	0.216	0.254	0.227	0.181	0.131
MacroPrep High Q	1.458	1.389	1.430	1.246	0.950	0.536	0.375	0.265	0.238	0.187	0.132	0.237
Nuvia Q	1.550	1.245	1.244	1.467	1.452	1.181	0.845	0.479	0.315	0.189	0.239	0.249
UnoSphere Q	1.517	1.258	0.908	1.187	1.093	0.752	0.455	0.246	0.130	0.080	0.065	0.106
ANX Sepharose 4FF High Sub	1.445	1.295	1.264	1.331	1.403	1.235	0.741	0.399	0.318	0.234	0.170	0.266
Capto DEAE	1.378	1.220	0.428	0.482	0.467	0.378	0.248	0.172	0.170	0.109	0.073	0.058
Capto Q	1.491	1.250	0.648	0.661	0.508	0.336	0.281	0.202	0.181	0.131	0.217	0.106
DEAE Sepharose FF	1.487	1.310	1.193	1.148	0.789	0.312	0.178	0.136	0.110	0.067	0.027	0.018
Q Sepharose XL	1.495	1.226	0.377	0.519	0.588	0.447	0.327	0.203	0.149	0.111	0.389	0.066
Source 30Q	1.501	1.384	1.315	1.117	0.596	0.244	0.162	0.115	0.107	0.119	0.068	0.062
Eshmuno Q	1.514	1.255	0.784	1.117	1.129	0.772	0.427	0.198	0.123	0.081	0.211	0.067
Fractogel EMD DEAE (M)	1.473	1.373	0.964	0.782	0.630	0.351	0.261	0.191	0.176	0.138	0.075	0.049
Fractogel EMD TMAE (M)	1.540	1.432	1.227	1.248	0.907	0.543	0.294	0.167	0.160	0.148	0.126	0.138
Fractogel EMD TMAE Hicap (M)	1.527	1.261	1.392	1.473	1.285	0.914	0.555	0.470	0.276	0.170	0.074	0.255
Hypercel Star AX	1.434	1.246	0.486	0.416	0.466	0.296	0.283	0.196	0.231	0.160	0.157	0.157
POROS 50D	1.528	1.377	1.341	0.998	0.510	0.271	0.231	0.184	0.170	0.130	0.370	0.094
POROS 50 HQ	1.537	1.442	1.510	1.405	1.264	0.891	0.588	0.366	0.304	0.201	0.150	0.442
POROS XQ	1.511	1.255	1.078	1.374	1.314	1.081	0.855	0.515	0.313	0.228	0.156	0.197
POROS 50 PI	1.550	1.414	0.925	0.785	0.771	0.599	0.470	0.283	0.261	0.214	0.121	0.288
TOYOPEARL NH2-750F	1.559	1.373	0.444	0.286	0.253	0.054	0.400	0.275	0.227	0.201	0.158	0.361
TOYOPEARL DEAE-650M	1.555	1.385	1.005	0.442	0.337	0.304	0.328	0.171	0.064	0.030	0.132	0.025
TOYOPEARL DEAE-650C	1.481	1.357	0.924	0.488	0.290	0.273	0.202	0.057	0.044	0.040	0.022	0.017
TOYOPEARL GigaCap Q-650M	1.500	1.216	1.338	1.372	1.222	0.935	0.690	0.436	0.274	0.173	0.160	0.184
TOYOPEARL GigaCap Q-650S	1.501	1.294	1.476	1.380	1.256	1.006	0.870	0.644	0.440	0.304	0.700	0.129
TOYOPEARL SuperQ-650C	1.497	1.264	1.210	1.109	0.757	0.431	0.275	0.194	0.176	0.168	0.130	0.076
TOYOPEARL SuperQ-650M	1.476	1.302	1.342	1.158	0.669	0.337	0.186	0.126	0.132	0.134	0.089	0.083
TOYOPEARL QAE 550C	1.220	0.637	0.582	0.794	0.654	0.571	0.483	0.304	0.218	0.148	0.079	0.112
TOYOPEARL Q-600C AR	1.457	1.226	0.560	0.790	1.043	0.863	0.510	0.255	0.155	0.104	0.075	0.101
TOYOPEARL GigaCap DEAE-650M	1.509	1.240	1.409	1.325	1.087	0.868	0.828	0.538	0.486	0.245	0.177	0.220

LEGEND: NR | LOW | HIGH

Figure S1. pH 7.0 (left) and pH 8.0 (right) Anion exchange Resin screen results. A280 (top) and Octet (bottom) results for each resin in the pH 7.0 (left) and pH 8.0 (right) anion exchange screen. Each row is subjected to a gradient with a green denoting high signal, orange denoting low signal, and grey indicating no data.

Figure S2

Resin	Flowthrough	Chase	100 mM NaCl	200 mM NaCl	300 mM NaCl	400 mM NaCl	500 mM NaCl	Strip
Macro-Prep High S	0.882	0.322	0.049	0.038	0.027	0.153	0.248	0.223
MacroCap SP	0.248	0.306	0.790	0.434	0.116	0.297	0.103	0.111
MacropPrep CM	0.910	0.342	0.053	0.041	0.035	0.028	0.026	0.023
Nuvia HR-S	0.222	0.182	0.628	0.751	0.129	0.297	0.121	0.164
Nuvia S	0.455	0.307	0.385	0.284	0.136	0.236	0.048	0.030
Capto S	0.820	0.285	0.098	0.168	0.100	0.071	0.033	0.029
Capto SP ImpRes	0.393	0.334	0.559	0.412	0.117	0.217	0.094	0.074
Source 30S	0.486	0.343	0.247	0.231	0.138	0.216	0.094	0.066
SP Sepharose FF	0.505	0.299	0.452	0.429	0.128	0.216	0.093	0.064
SP Sepharose XL	0.732	0.280	0.138	0.175	0.101	0.112	0.054	0.038
CM Sepharose HP	0.550	0.313	0.150	0.242	0.182	0.351	0.065	0.033
Eshmuno CPX	0.212	0.124	0.056	0.383	0.555	0.527	0.126	0.242
Eshmuno S	0.526	0.255	0.208	0.270	0.120	0.134	0.062	0.046
Fractogel EMD COO-	0.360	0.393	0.513	0.268	0.099	0.178	0.083	0.135
Fractogel EMD SE Hicap (M)	0.222	0.126	0.268	1.214	0.150	0.219	0.076	0.070
Fractogel EMD SO3-	0.234	0.136	0.080	0.468	0.542	0.486	0.130	0.253
POROS 50 HS	0.212	0.118	0.056	0.622	0.426	0.401	0.110	0.251
POROS XS	0.211	0.112	0.052	0.427	0.464	0.400	0.088	0.213
Toyopearl CM-650C	0.615	0.297	0.134	0.242	0.149	0.269	0.080	0.040
Toyopearl CM-650M	0.613	0.321	0.152	0.281	0.156	0.276	0.069	0.031
Toyopearl GigaCap CM-650M	0.456	0.218	0.335	0.335	0.134	0.198	0.080	0.040
Toyopearl GigaCap S-650M	0.519	0.267	0.285	0.274	0.142	0.165	0.069	0.030
Toyopearl MegaCap II SP-550EC	0.785	0.290	0.130	0.213	0.108	0.128	0.072	0.026
Toyopearl MX-Trp-650M	0.349	0.171	0.096	0.186	0.138	0.360	0.151	0.293
Toyopearl SP-550C	0.333	0.168	0.109	0.530	0.320	0.459	0.112	0.237
Toyopearl SP-650C	0.607	0.304	0.181	0.321	0.143	0.271	0.054	0.028
Toyopearl SP-650M	0.571	0.301	0.178	0.323	0.143	0.314	0.054	0.030
S Ceramic HyperD 20	0.526	0.367	0.178	0.198	0.099	0.169	0.061	0.049
Praesto SP45	0.331	0.314	0.701	0.449	0.116	0.244	0.092	0.077
Praesto SP65	0.444	0.326	0.578	0.384	0.118	0.240	0.089	0.071
Praesto SP90	0.532	0.282	0.471	0.398	0.132	0.214	0.091	0.062
Eshmuno CPX	0.232	0.143	0.060	0.445	0.591	0.492	0.131	0.252

Average	Standard Deviation	Median
0.249	0.193	0.212

Resin	Flowthrough	Chase	100 mM NaCl	200 mM NaCl	300 mM NaCl	400 mM NaCl	500 mM NaCl	Strip
Macro-Prep High S	0.960	0.413	0.089	0.071	0.051	0.072	0.044	0.041
MacroCap SP	0.815	0.397	0.160	0.379	0.112	0.120	0.049	0.035
MacropPrep CM	0.964	0.301	0.048	0.080	0.059	0.051	0.041	0.050
Nuvia HR-S	0.753	0.366	0.258	0.422	0.144	0.132	0.052	0.050
Nuvia S	0.961	0.280	0.105	0.136	0.038	0.027	0.024	0.022
Capto S	1.035	0.254	0.050	0.045	0.025	0.027	0.022	0.023
Capto SP ImpRes	0.797	0.355	0.143	0.315	0.152	0.151	0.084	0.123
Source 30S	0.942	0.342	0.124	0.259	0.096	0.073	0.045	0.041
SP Sepharose FF	0.830	0.338	0.147	0.324	0.142	0.139	0.074	0.072
SP Sepharose XL	0.977	0.271	0.089	0.124	0.044	0.038	0.026	0.023
CM Sepharose HP	0.916	0.409	0.137	0.200	0.039	0.028	0.022	0.021
Eshmuno CPX	0.427	0.180	0.363	1.332	0.212	0.180	0.095	0.286
Eshmuno S	0.960	0.220	0.069	0.157	0.056	0.038	0.028	0.024
Fractogel EMD COO-	0.891	0.339	0.105	0.248	0.072	0.043	0.029	0.025
Fractogel EMD SE Hicap (M)	0.635	0.325	0.641	0.533	0.121	0.151	0.060	0.036
Fractogel EMD SO3-	0.440	0.193	0.274	1.365	0.219	0.177	0.094	0.311
POROS 50 HS	0.419	0.198	0.603	1.113	0.150	0.144	0.094	0.299
POROS XS	0.403	0.180	0.421	1.148	0.185	0.169	0.055	0.234
Toyopearl CM-650C	0.968	0.368	0.074	0.102	0.035	0.026	0.023	0.025
Toyopearl CM-650M	0.972	0.357	0.069	0.072	0.029	0.023	0.028	0.024
Toyopearl GigaCap CM-650M	0.939	0.246	0.088	0.179	0.053	0.032	0.051	0.022
Toyopearl GigaCap S-650M	1.004	0.261	0.078	0.081	0.031	0.027	0.041	0.022
Toyopearl MegaCap II SP-550EC	0.987	0.288	0.078	0.082	0.032	0.028	0.046	0.021
Toyopearl MX-Trp-650M	0.769	0.256	0.135	0.396	0.181	0.187	0.102	0.193
Toyopearl SP-550C	0.562	0.259	0.501	0.701	0.168	0.175	0.106	0.224
Toyopearl SP-650C	0.969	0.374	0.078	0.087	0.030	0.027	0.025	0.023
Toyopearl SP-650M	0.976	0.381	0.094	0.096	0.029	0.026	0.023	0.024
S Ceramic HyperD 20	0.976	0.357	0.063	0.093	0.046	0.038	0.032	0.035
Praesto SP45	0.802	0.385	0.162	0.343	0.137	0.142	0.073	0.070
Praesto SP65	0.830	0.375	0.152	0.345	0.139	0.123	0.058	0.045
Praesto SP90	0.826	0.332	0.151	0.300	0.144	0.128	0.072	0.069
Eshmuno CPX	0.435	0.198	0.410	1.396	0.213	0.178	0.099	0.296

Average	Standard Deviation	Median
0.254	0.300	0.138

Resin	Flowthrough	Chase	100 mM NaCl	200 mM NaCl	300 mM NaCl	400 mM NaCl	500 mM NaCl	Strip
Macro-Prep High S	43.260	49.790	<LLOQ	<LLOQ	<LLOQ	<LLOQ	0.000	0.000
MacroCap SP	82.980	94.305	87.885	61.075	<LLOQ	1.830	<LLOQ	<LLOQ
MacropPrep CM	51.805	46.940	<LLOQ	<LLOQ	<LLOQ	<LLOQ	<LLOQ	<LLOQ
Nuvia HR-S	44.285	<LLOQ	89.600	80.180	<LLOQ	3.130	<LLOQ	<LLOQ
Nuvia S	84.270	84.600	67.055	0.000	<LLOQ	0.000	<LLOQ	<LLOQ
Capto S	58.540	45.790	<LLOQ	<LLOQ	<LLOQ	<LLOQ	<LLOQ	<LLOQ
Capto SP ImpRes	64.200	81.035	91.355	69.045	<LLOQ	0.000	<LLOQ	<LLOQ
Source 30S	93.125	92.660	79.530	18.285	<LLOQ	4.390	<LLOQ	<LLOQ
SP Sepharose FF	51.805	78.550	85.770	64.210	<LLOQ	0.000	<LLOQ	<LLOQ
SP Sepharose XL	63.900	68.485	<LLOQ	<LLOQ	<LLOQ	<LLOQ	<LLOQ	<LLOQ
CM Sepharose HP	86.795	72.545	<LLOQ	3.815	<LLOQ	1.145	<LLOQ	<LLOQ
Eshmuno CPX	<LLOQ	<LLOQ	<LLOQ	81.160	77.990	47.075	<LLOQ	11.715
Eshmuno S	81.175	72.725	<LLOQ	50.990	<LLOQ	<LLOQ	<LLOQ	<LLOQ
Fractogel EMD COO-	74.300	91.465	94.795	52.480	<LLOQ	<LLOQ	<LLOQ	<LLOQ
Fractogel EMD SE Hicap (M)	0.330	<LLOQ	93.480	83.730	<LLOQ	0.000	<LLOQ	<LLOQ
Fractogel EMD SO3-	8.485	<LLOQ	<LLOQ	79.990	83.760	42.100	<LLOQ	16.980
POROS 50 HS	<LLOQ	<LLOQ	<LLOQ	83.160	76.855	27.780	<LLOQ	3.555
POROS XS	<LLOQ	<LLOQ	<LLOQ	85.650	74.920	35.860	<LLOQ	14.470
Toyopearl CM-650C	80.960	61.485	<LLOQ	8.245	<LLOQ	1.455	<LLOQ	<LLOQ
Toyopearl CM-650M	72.095	58.135	<LLOQ	4.695	<LLOQ	0.800	<LLOQ	<LLOQ
Toyopearl GigaCap CM-650M	89.720	83.135	77.270	44.330	<LLOQ	<LLOQ	<LLOQ	<LLOQ
Toyopearl GigaCap S-650M	85.310	72.180	52.250	0.000	<LLOQ	<LLOQ	<LLOQ	<LLOQ
Toyopearl MegaCap II SP-550EC	57.310	57.950	<LLOQ	1.235	<LLOQ	<LLOQ	<LLOQ	<LLOQ
Toyopearl MX-Trp-650M	89.460	<LLOQ	<LLOQ	<LLOQ	<LLOQ	90.180	<LLOQ	70.685
Toyopearl SP-550C	63.740	<LLOQ	<LLOQ	83.430	76.505	44.780	<LLOQ	7.280
Toyopearl SP-650C	80.920	72.105	<LLOQ	2.495	<LLOQ	1.185	<LLOQ	<LLOQ
Toyopearl SP-650M	84.285	78.995	<LLOQ	2.390	<LLOQ	1.485	<LLOQ	<LLOQ
S Ceramic HyperD 20	88.825	81.365	<LLOQ	<LLOQ	<LLOQ	<LLOQ	<LLOQ	<LLOQ
Praesto SP45	61.720	89.455	88.520	63.580	<LLOQ	0.000	<LLOQ	<LLOQ
Praesto SP65	53.330	88.310	86.740	80.850	<LLOQ	0.000	<LLOQ	<LLOQ
Praesto SP90	47.840	76.105	90.410	87.580	<LLOQ	0.000	<LLOQ	<LLOQ
Eshmuno CPX	0.000	<LLOQ	<LLOQ	78.595	77.980	45.340	<LLOQ	8.180

Resin	Flowthrough	Chase	100 mM NaCl	200 mM NaCl	300 mM NaCl	400 mM NaCl	500 mM NaCl	Strip
Macro-Prep High S	41.195	44.240	<LLOQ	<LLOQ	<LLOQ	<LLOQ	<LLOQ	<LLOQ
MacroCap SP	53.860	46.920	26.200	5.555	<LLOQ	<LLOQ	<LLOQ	<LLOQ
MacropPrep CM	45.865	41.100	<LLOQ	<LLOQ	<LLOQ	<LLOQ	<LLOQ	<LLOQ
Nuvia HR-S	53.930	52.070	46.490	8.525	0.655	<LLOQ	<LLOQ	<LLOQ
Nuvia S	45.440	37.895	<LLOQ	<LLOQ	<LLOQ	<LLOQ	<LLOQ	<LLOQ
Capto S	43.035	38.390	<LLOQ	<LLOQ	<LLOQ	<LLOQ	<LLOQ	<LLOQ
Capto SP ImpRes	55.400	50.160	21.010	7.000	2.110	1.510	<LLOQ	<LLOQ
Source 30S	48.520	42.250	<LLOQ	3.175	<LLOQ	<LLOQ	<LLOQ	<LLOQ
SP Sepharose FF	54.880	45.005	23.565	2.630	1.210	0.645	<LLOQ	<LLOQ
SP Sepharose XL	46.360	37.630	<LLOQ	<LLOQ	<LLOQ	<LLOQ	<LLOQ	<LLOQ
CM Sepharose HP	46.975	37.675	<LLOQ	1.305	<LLOQ	<LLOQ	<LLOQ	<LLOQ
Eshmuno CPX	8.560	15.565	77.345	74.280	7.790	3.420	<LLOQ	5.390
Eshmuno S	46.080	36.685	<LLOQ	2.240	<LLOQ	<LLOQ	<LLOQ	<LLOQ
Fractogel EMD COO-	47.735	41.630	<LLOQ	5.420	<LLOQ	<LLOQ	<LLOQ	<LLOQ
Fractogel EMD SE Hicap (M)	43.730	50.710	73.205	22.810	<LLOQ	2.225	<LLOQ	<LLOQ
Fractogel EMD SO3-	9.605	19.440	61.265	73.265	7.600	4.265	<LLOQ	2.200
POROS 50 HS	11.855	25.195	80.850	65.630	3.370	5.240	<LLOQ	3.950
POROS XS	10.820	19.425	81.355	74.035	7.105	3.575	<LLOQ	4.225
Toyopearl CM-650C	44.610	38.790	<LLOQ	<LLOQ	<LLOQ	<LLOQ	<LLOQ	<LLOQ
Toyopearl CM-650M	43.275	37.920	<LLOQ	<LLOQ	<LLOQ	<LLOQ	<LLOQ	<LLOQ
Toyopearl GigaCap CM-650M	54.220	38.385	<LLOQ	1.530	<LLOQ	<LLOQ	<LLOQ	<LLOQ
Toyopearl GigaCap S-650M	43.145	38.635	<LLOQ	<LLOQ	<LLOQ	<LLOQ	<LLOQ	<LLOQ
Toyopearl MegaCap II SP-550EC	41.830	38.685	<LLOQ	<LLOQ	<LLOQ	<LLOQ	<LLOQ	<LLOQ
Toyopearl MX-Trp-650M	55.610	44.935	<LLOQ	38.965	15.290	3.700	<LLOQ	0.040
Toyopearl SP-550C	47.940	51.115	79.975	57.325	8.015	2.465	<LLOQ	1.480
Toyopearl SP-650C	45.295	38.010	<LLOQ	<LLOQ	<LLOQ	<LLOQ	<LLOQ	<LLOQ
Toyopearl SP-650M	49.270	37.940	<LLOQ	<LLOQ	<LLOQ	<LLOQ	<LLOQ	<LLOQ
S Ceramic HyperD 20	45.105	41.860	<LLOQ	<LLOQ	<LLOQ	<LLOQ	<LLOQ	<LLOQ
Praesto SP45	56.060	47.720	22.025	4.450	<LLOQ	1.340	<LLOQ	<LLOQ
Praesto SP65	53.380	45.300	17.385	6.395	1.200	<LLOQ	<LLOQ	<LLOQ
Praesto SP90	53.700	45.480	22.365	6.690	0.925	<LLOQ	<LLOQ	<LLOQ
Eshmuno CPX	7.550	15.660	76.570	73.530	5.945	4.190	<LLOQ	4.100

LEGEND			
<LLOQ	LOW		HIGH

Figure S2. Cation exchange Resin screen results. A280 (top) and GXII (bottom) results for each resin in the pH 4,0 (left) and pH 5.0 (right) cation exchange screen. Each row is subjected to a gradient with a green denoting high signal, orange denoting low signal, and grey indicating no data. The GXII results for fractions which had an A280 signal greater than the median A280 signal are reported.

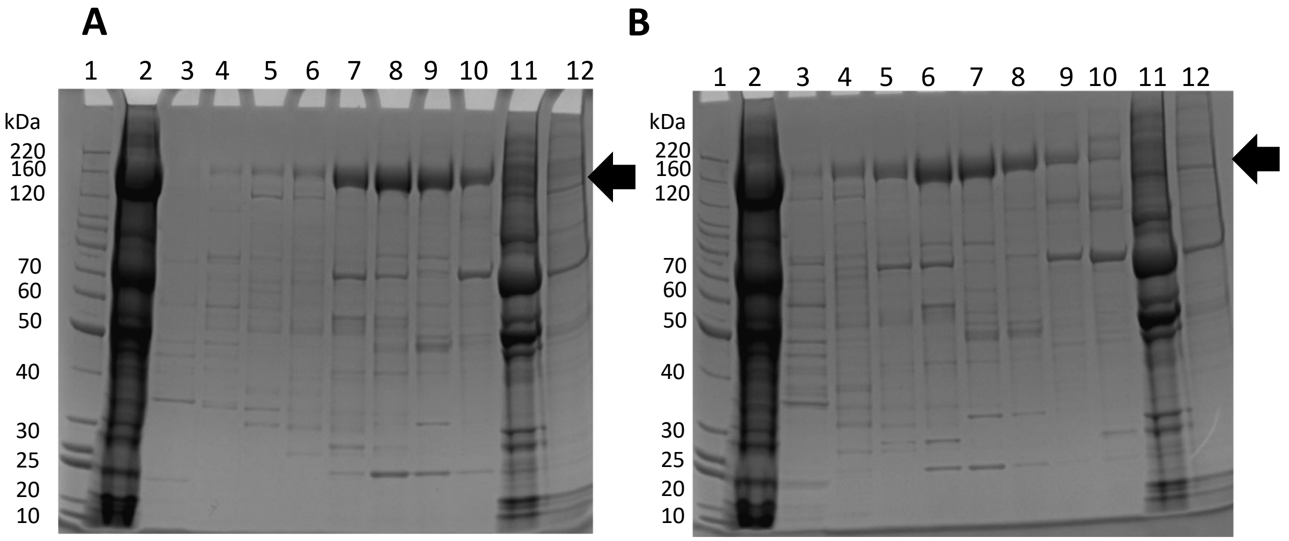

Figure S3. AEX Capture Step Buffer Selection SDS-PAGE. 25 mM MES pH 6.5 running buffer (A) and 25 mM Sodium Phosphate, pH 6.5 buffer (B), where well 1 is the benchmark protein ladder, well 2 is the TFF retentate (POROS 50 D load), well 3 is the flowthrough fraction, wells 4 through 11 are wash fractions in running buffer with 50 mM NaCl (4), 75 mM NaCl (5), 100 mM NaCl (6), 125 mM NaCl (7), 150 mM NaCl (8), 175 mM NaCl (9), 200 mM NaCl (10), and 500 mM NaCl (11), and the 1M NaCl strip in running buffer is well 12.

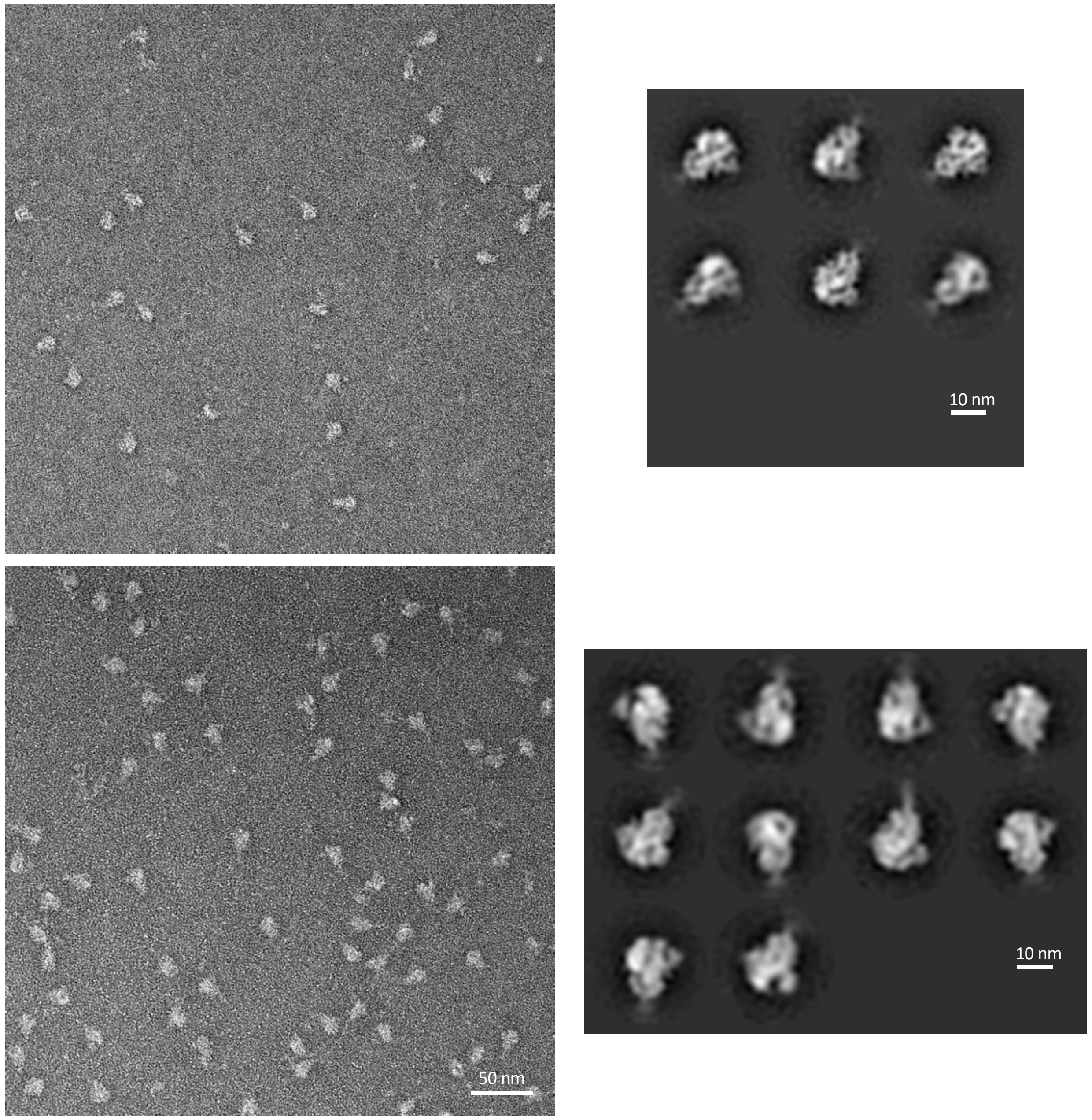

Figure S4. Cation Exchange proof of concept NS-EM. Top row: SP650M flow through. Bottom row: Nuvia HR-S elution. Left column: representative images at 100,000x. Right column: 2D Classes.

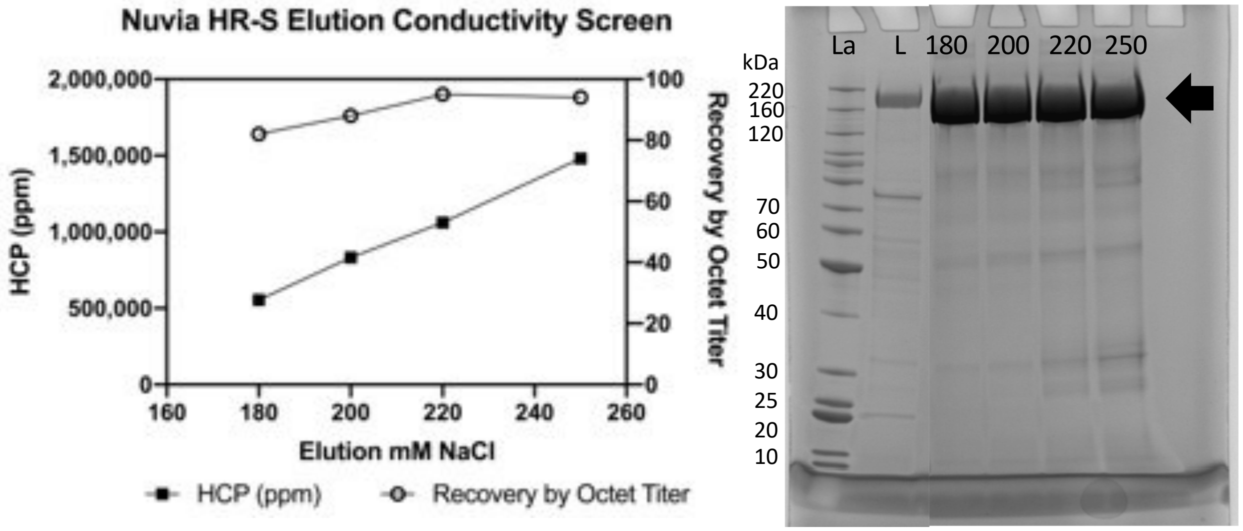

Figure S5. Nuvia HR-S Elution Buffer Selection. Left: HCP (ppm) (closed square, left axis) and Recovery by Octet Titer (open circle, right axis) are plotted against Elution mM NaCl ranging from 180 mM NaCl to 250 mM NaCl. Right: Samples are assessed by SDS-PAGE. La: ladder, L: load; numbers represent mM NaCl elution.

Unit Operation	Operation Step	Purification Process
Clarification	Feed	6.5 L Cell culture harvest
	Filter Train	0.11 m^2 Clarisolve 20MS, 0.11 m^2 Millistak+ F0HC, 0.26 m^2 0.8/0.2 µm sterile filter
	Flux	60 LMH (based on F0HC area)
TFF1	Load Material	Clarified cell culture harvest
	Filter area	0.5 m^2
	MWCO	300 kDa
	Feed flux	330 LMH
	TMP	10 psi
	Equilibration buffer	25 mM MES, 25 mM NaCl pH 6.5
	Ultrafiltration and Diafiltration	5X/5X
	Diafiltration buffer	25 mM MES, 25 mM NaCl pH 6.5
AEX 1	Resin	POROS 50 D
	Load Material	TFF1 Retentate & chase pool, 0.8/0.2 µm filtered
	Loading capacity	20-25 mg/mL-r (total protein by A280)
	Equilibration buffer	25 mM MES, 25 mM NaCl pH 6.5
	Wash buffer	25 mM MES, 100 mM NaCl pH 6.5
	Elution buffer	25 mM MES, 190 mM NaCl pH 6.5
Low pH treatment	Conditioning solution	5 N HCl
	Hold time	60 minutes
CEX 1	Resin	Toyopearl SP-650M
	Load Material	Conditioned, low pH treated capture step eluate
	Conditioning solution	50 mM Sodium Citrate pH 4.0
	Loading capacity	< 15 mg/mL-r (total protein by A280)
	Equilibration & chase buffer	50 mM Sodium Citrate, 50 mM NaCl pH 4.0
CEX 2	Resin	Nuvia HR-S
	Load Material	SP-650M flow through and chase
	Loading capacity	30 mg/mL-r (total protein by A280)
	Equilibration buffer	50 mM Sodium Citrate, 50 mM NaCl pH 4.0
	Wash buffer	50 mM Sodium Citrate, 50 mM NaCl pH 4.0
	Elution buffer	50 mM Sodium Citrate, 180 mM NaCl pH 4.0
20 nm Filtration	Load Material	Nuvia HR-S eluate
	Pre-Filter	Viresolve Shield
	20 nm Filter	Viresolve Pro
	Loading capacity	< 10 g/m^2
TFF2	Load Material	20 nm filtrate
	Loading capacity	15 g/m^2
	Feed flux	300 LMH
	MWCO	100 kDa
	TMP	7.3 psi
	Equilibration buffer	50 mM Sodium Citrate, 180 mM NaCl pH 4.0
	Diafiltration buffer	10 mM Histidine, 150 mM NaCl, 5% (w/v) Sucrose pH 5.5
	Ultrafiltration and Diafiltration	2X./20X

Table S1. Process Parameters. Specific process parameters for each unit operation of the HexaPro proof of concept are listed.
